# Bridging Blood and Skin: Biomarker Profiling in Dermal Interstitial Fluid (dISF) for Minimally Invasive Diagnostics

**DOI:** 10.3390/bios15050301

**Published:** 2025-05-09

**Authors:** Yann Sprunger, Johan Longo, Ali Saeidi, Adrian M. Ionescu

**Affiliations:** 1 Xsensio SA, 1015 Lausanne, Switzerland; johan.longo@xsensio.com (J.L.); info@xsensio.com (A.S.); 2 Nanoelectronic Devices Laboratory, Ecole Polytechnique Fédérale de Lausanne (EPFL), 1015 Lausanne, Switzerland; adrian.ionescu@epfl.ch

**Keywords:** dermal interstitial fluid (dISF), minimally invasive diagnostics, microneedle biosampling, biomarker profiling, serum to ISF correlation

## Abstract

Understanding the biochemical relationship between serum and dermal interstitial fluid (dISF) is critical for advancing minimally invasive diagnostics with wearables and point of care devices focusing on most relevant biomarkers accessible in the ISF. This work compares the composition of dISF and serum using Xsensio’s microneedle-based collector, which yields an average of 3.4 μL/h. In the first study, total protein content, human serum albumin (HSA), and immunoglobulin G (IgG) are quantified in twelve volunteers. A second study is dedicated to screening 50 inflammation-related protein biomarkers across twenty volunteers. The results demonstrate that dISF closely resembles serum in its major protein constituents but at reduced concentrations (e.g., 57% for total protein). Strong correlations are observed between dISF and serum for CRP and SAA ($R^{2}>0.87$), primarily driven by a subject with pathological levels, demonstrating the ability of dISF to reflect systemic inflammation. This study originally reports NT-proBNP detection at comparable levels in both fluids, suggesting that dISF could serve as a reliable proxy for blood NT-proBNP in the non-invasive diagnosis of cardiac failure. Cytokine profiling reveals 36 detectable cytokines, including several unique to dISF. Notably, interleukin concentrations are found to be highly similar between the two fluids. These experimental findings support dISF as a promising diagnostic medium for monitoring both localized and systemic biomarkers in clinical applications.

## 1. Introduction

Interstitial fluid (ISF) surrounds cells in tissues, acting as a medium for nutrient delivery, waste removal, and molecular signaling. The human body contains at least three times more ISF than blood, offering significant diagnostic potential that remains underexplored [1,2]. Dermal ISF (dISF), located within the skin, is thought to share a biomarker composition similar to blood. Positioned near the skin’s surface, dISF presents strong opportunities for minimally invasive sampling and continuous biomarker monitoring.

In recent years, dISF has gained attention as a promising matrix for continuous monitoring, inspired by the success of continuous glucose monitors (CGMs) in diabetes management. CGMs measure glucose levels in dISF, providing real-time, continuous data without frequent blood sampling [3,4]. Their success underscores dISF’s potential for wearable devices to monitor additional biomarkers, paving the way for real-time health tracking, personalized medical interventions, and innovative diagnostic technologies.

ISF holds significant diagnostic potential but remains underexplored in clinical settings, partly due to sampling difficulties and the resulting limited knowledge of its composition. In the skin, ISF resides in the dermis, comprising approximately 70% of tissue volume, yet its collection is challenging [5]. Current methods are often invasive, uncomfortable, or impractical. Biopsies, though effective, are painful, require expertise, and risk scarring, while producing mixed samples of extracellular and intracellular components [6]. Suction blisters, requiring prolonged suction and heat, can damage the skin and introduce contamination [7]. Techniques such as microdialysis and open-flow microperfusion are time-consuming, invasive, and restricted by membrane permeability [8,9]. Reverse iontophoresis, which relies on electric currents, is limited to small molecules, can irritate the skin, and requires frequent calibration [10]. In contrast, microneedle-based methods—the approach used in this study—are nearly non-invasive and provide high-quality, non-diluted dISF, although they can collect only a few microliters per hour [5,11,12]. These limitations have hindered comprehensive ISF characterization and its integration into diagnostic applications.

Despite these challenges, pioneering studies have explored the composition of ISF [5,11,12], some focusing specifically on cytokines and other inflammatory markers [13,14,15]. For example, a comparative study by Sjöbom et al. investigated inflammatory markers in suction blister fluid (SBF) and plasma from healthy volunteers, providing insights into ISF’s potential role in inflammation monitoring [13]. Yiran Li et al. examined cytokine patterns in the blister fluid and plasma of patients with fracture blisters [14]. Moneidero et al. characterized inflammatory mediators and the metabolome in patients with atopic dermatitis using dermal open-flow microperfusion, further highlighting dISF’s role in skin-related conditions [15]. Additionally, studies have examined the dynamics of lactate and other metabolites in dISF, emphasizing its responsiveness to physiological changes [16,17,18]. However, despite these foundational studies, there remains a gap in understanding the primary components of dISF and establishing a baseline cytokine profile in healthy individuals. A comprehensive characterization of dISF in healthy subjects is essential to provide a reference for future studies and to explore its potential for broader clinical applications.

In this work, we aim to address this gap by shedding light on the composition of dISF. In an initial study, we collected dISF and serum from twelve healthy volunteers. We measured the total protein content in both matrices, focusing on key constituents such as human serum albumin (HSA) and immunoglobulin G (IgG). In a second study, we investigated the baseline inflammatory profile of dISF and its correlation with blood. dISF and serum were collected from twenty healthy volunteers and analyzed using both Meso Scale Discovery (MSD) and Proximity Extension Assay (PEA) technologies to screen over fifty inflammatory markers. This work aims to establish a foundational understanding of dISF composition, providing a reference for future research and a basis for exploring its use in continuous biomarker monitoring.

## 2. Materials and Methods

### 2.1. Disf Collector, Skin Damage Assessment and Study Plan

Twelve volunteers (six males and six females, aged 20–45 years) were recruited to measure human serum albumin (HSA), immunoglobulin G (IgG), and total protein content in both dermal interstitial fluid (dISF) and serum. Blood samples were collected via finger prick using a lancet and stored in BD^TM^ Microtainer tubes (Becton, Dickinson and Company, Franklin Lakes, NJ, USA) for 30 min before being centrifuged for two minutes to obtain serum.

**Figure 1:**
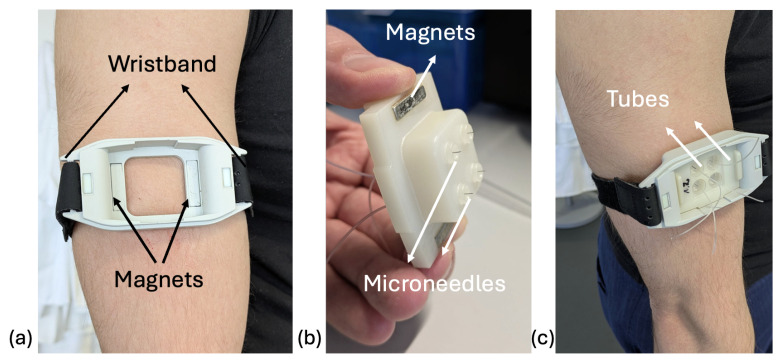
Xsensio’s dermal interstitial fluid (dISF) collector. (**a**) Base component of the dISF collector, serving as a support structure with a wristband on both sides to apply pressure and embedded magnets to attach to the collection component. (**b**) Collection component featuring BD^TM^ 31G microneedles connected to Tygon^TM^ tubes for dISF extraction. (**c**) Example of dISF collection on the biceps–triceps region. Tygon^TM^ tubes are connected to the microneedles to extract the fluid.

dISF was collected using Xsensio’s in-house interstitial fluid collector, which consists of a base with four circular openings, each fitted with a certified BD^TM^ 5 mm microneedle 31 Gauge (31G). A wristband is attached to both sides of the base to apply pressure (Figure 1a,c). Magnets are embedded in the base to facilitate attachment to the collection component. Tygon^TM^ tubing (Saint-Gobain Performance Plastics, Akron, OH, USA) is directly connected to the microneedles (Figure 1b,c) and dISF is collected by drawing the fluid from the Tygon^TM^ tube into an Eppendorf^TM^ tube (Eppendorf SE, Hamburg, Germany) using a pipette. The collector is autoclaved before use, and the skin is disinfected with isopropanol prior to microneedle insertion. The collection time for dISF was approximately one hour. The collector was placed on the biceps–triceps region (Figure 1c).

Volunteers were not asked to fast, and no information regarding health status was collected (except for pregnancy, which was an exclusion criterion). Both dISF and serum samples were stored at −20 °C until analysis.

To assess the risk of skin damage, a morphological evaluation was conducted using non-invasive techniques. The skin of four volunteers who wore the collector for one hour was examined by optical coherence tomography (OCT) 48 h after collection. OCT is a cutting edge imaging technology that uses light instead of sound to capture detailed cross sectional images of tissue. Much like ultrasound employs sound waves, the VivoSight^TM^ (Michelson Diagnostics Ltd., Maidstone, Kent, UK) OCT system utilizes eye-safe infrared laser light to generate high resolution 3D image blocks, with spatial precision finer than 10 μm. With an ultra-wide field of view of 6 mm × 6 mm and a depth of 1 mm, the user is not only able to see deep into the dermis but also get a full picture of the sub-surface microstructure (https://vivosight.com/).

### 2.2. Total Protein Content, HSA, and IgG Quantification

The Thermo Scientific™ Pierce™ BCA Protein Assay Kit (Thermo Fisher Scientific Inc., Waltham, MA, USA), a detergent-compatible formulation based on bicinchoninic acid (BCA) for the colorimetric detection and quantitation of total protein, was used. The dillution factor was 100 for serum and 50 for dISF, in PBS 1X. The Human Albumin (ALB) ELISA Kit from Invitrogen^TM^ (Thermo Fisher Scientific Inc., Waltham, MA, USA) was used to detect and quantify the level of human albumin in serum and dISF. The dilution factor used was both 500,000 for dISF and serum. Human IgG ELISA kit from ICL^TM^ (Immunology Consultants Laboratory, Inc., Portland, OR, USA) was used to quantify IgG. The dilution factor used was both 80,000 for dISF and serum in the working solution.

### 2.3. Meso Scale Discovery (MSD)

The V-plex vascular injury panel II MSD^TM^ method was used to quantify serum amyloid A (SAA), C-reactive protein (CRP), vascular cell adhesion molecule 1 (VCAM) and intercellular adhesion molecule 1 (ICAM) both in dISF and serum. The dilution factor for the V-plex vasculary injury panel II was 1000. A singleplex assay (R-plex) was used to quantify NT-pBNP, with the same method, using a dilution factor of 25. The method is a sandwich enzyme multiplex immunoassay with electrochemiluminescence detection via MSD tag label, that emits light at 620 nm upon electrochemical stimulation initiated at the electrode surface of MSD microplates.

### 2.4. Olink 48 Target Cytokine Panel

Proteins were quantified using the Olink^®^ Target 48 Cytokine Panel (Olink Proteomics AB, Uppsala, Sweden) following the manufacturer’s guidelines. This protocol employs PEA (Proximity Extension Assay) technology, which allows for the simultaneous analysis of 45 analytes using just 1 μL of sample. Briefly, pairs of antibody probes labeled with oligonucleotides bind to their target proteins. When the probes come into close proximity, their oligonucleotides hybridize pairwise. The subsequent addition of DNA polymerase triggers a proximity-dependent DNA polymerization event, generating a unique PCR target sequence. This DNA sequence is then detected and quantified using real-time PCR. The resulting data undergo quality control and normalization through internal and external controls. The final output is presented as Normalized Protein Expression (NPX) values, an arbitrary unit on a log2 scale where higher values indicate greater protein expression.

## 3. Results

### 3.1. dISF Collector Performance

**Figure 2:**
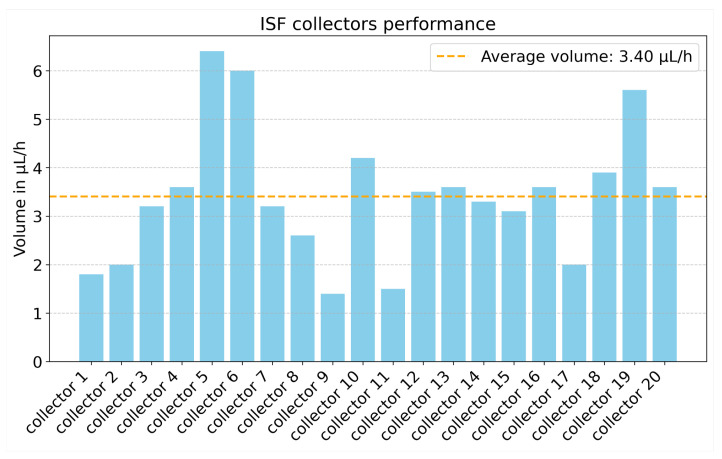
dISF collection performance in one hour for 20 collections. An average volume of 3.4 μL of clear dISF was obtained.

On a set of twenty collections, an average volume of 3.4 μL per hour was obtained. (Figure 2). A significant degree of inter-individual variability was observed, with 3 subject subjects yielding over 5 μL and 5 subjects getting less than 2 μL. Furthermore, the distribution of collected dISF within the Tygon^TM^ tubing was inconsistent. In some cases, all four tubes collected comparable volumes, while in others, fluid was collected in only a single tube. Blood contamination was observed in approximately 20% of collections. When contamination was detected, the entire tube was discarded to avoid sample compromise. The biceps–triceps region was established as the most effective site, offering both a high yield of dISF and greater ease of use while maintaining subject comfort.

### 3.2. OCT Skin Damage Assessment

**Figure 3:**
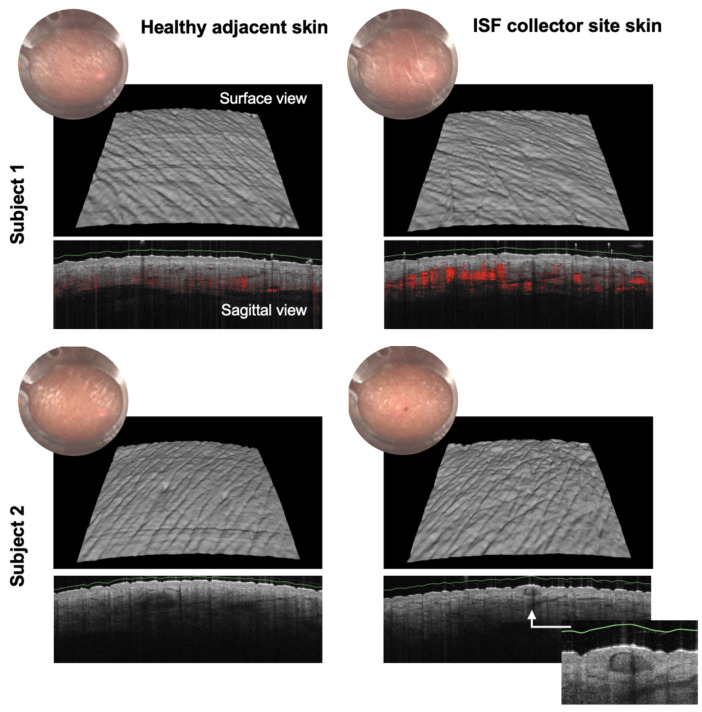
Optical Coherence Tomography (OCT) for Skin Damage Assessment: four volunteers wore the dISF collector for one hour and were examined 48 h later using OCT. No visible changes were detected in the epidermis, suggesting full recovery without scarring.

OCT imaging was performed on the collection site as well as on adjacent skin, providing an internal control. Morphological observations (Figure 3) revealed the presence of a longitudinal supra-epidermal crust (scratch) and an intra-epidermal crust (spot) at the collection site. These subtle morphological changes confirm that the OCT imaging was conducted precisely at the microneedle insertion site. Importantly, these changes did not extend beyond the epidermis, indicating that full recovery without scarring is expected. Dermatological assessment concludes that one-hour dISF collection using Xsensio’s collector leaves the skin essentially intact from a microscopic morphological perspective.

### 3.3. Albumin, Immunoglobulin G (IgG) and Total Protein

In most physiology textbooks, the total protein content in serum is reported to range from 60 to 80 g/L, with 50–60% consisting of albumin, 30–40% of globulins inluding 10–20% of immunoglobulin G (IgG) [19,20]. However, there is limited information on these proportions in dermal interstitial fluid (dISF). To compare the main constituents of dISF and serum, we conducted a BCA assay to measure the total protein content, and two ELISAs to quantify human serum albumin (HSA) and IgG concentrations.

**Figure 4:**
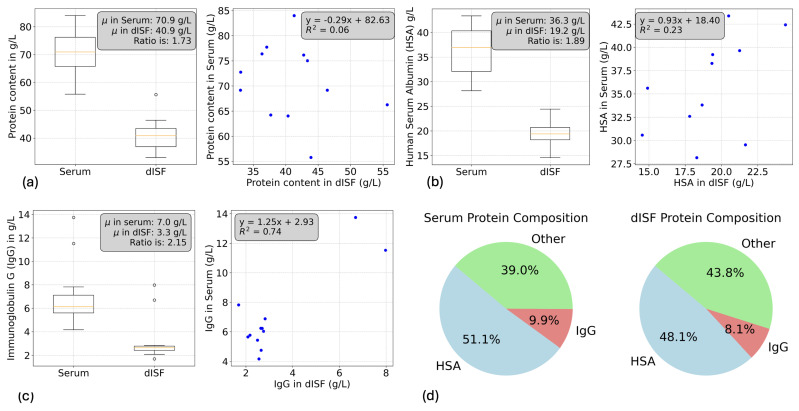
Comparison of main physiological constituents between serum and dISF. (**a**) Total protein content shows lower levels in dISF (serum-to-dISF ratio: 1.73) with weak correlation ($R^{2}=0.06$). (**b**) Human serum albumin (HSA) shows a ratio of 1.89 with moderate correlation ($R^{2}=0.23$). (**c**) Immunoglobulin G (IgG) concentration displays a serum-to-dISF ratio of 2.15 and a strong correlation ($R^{2}=0.74$). (**d**) Protein composition is similar between serum and dISF, as illustrated by pie charts.

The total protein content in serum ranged from 55 to 85 g/L, with an average of 70.89 g/L (Figure 4a), while in dISF, it ranged from 32 to 55 g/L, with an average of 40.92 g/L, resulting in a serum-to-dISF ratio of 1.73. IgG content ranged from 4 to 14 g/L in serum and 1.5–8 g/L in dISF, with an average serum-to-dISF ratio of 2.15 (Figure 4c). HSA content ranged from 28 to 44 g/L in serum and 14–24 g/L in dISF, yielding a serum-to-dISF ratio of 1.89 (Figure 4b). We observed one outlier in the dISF total protein content (55 g/L, 1.4 times higher than the average) and two outliers in the IgG measurements for both serum and dISF. By averaging the individual compositions of each subject, we calculated proportions of 51.1% HSA and 9.9% IgG in serum, and 48.1% HSA and 8.1% IgG in dISF (Figure 4d).

The average protein content in serum aligns with values established in the literature. With average concentrations of 36.30 g/L for HSA and 7.02 g/L for IgG, the values are slightly lower than some reported in the literature but remain within physiological ranges. However, a significant number of individual samples fell below the lower limit of the physiological range for both proteins. The calibration curves and assay controls were within the expected range, and we currently do not have a clear explanation for these lower values. The serum proportions are consistent with the lower boundary of textbook values (50% HSA and 10% IgG). Interestingly, dISF showed similar protein proportions, with dISF/serum average ratios of 57% for total protein, 53% for HSA, and 47% for IgG. These values corroborate the hypothesis that ISF is an ultrafiltrate of plasma with protein concentrations between 50% and 60% of plasma levels [21,22].

However, there is limited correlation between protein concentrations in serum and dISF. With R^2^ values of 0.06 for total protein content and 0.23 for HSA, we found no significant correlation between serum and dISF levels for these proteins; only IgG showed a strong correlation (R^2^ of 0.74), primarily influenced by two high-value outliers. The samples were assessed in duplicates, with a dispersion below 5%, ruling out measurement artifacts. While chronic infections, autoimmune diseases, or recent vaccinations could potentially explain these elevated values, they remain within non-pathological ranges. The concentration of proteins in ISF is affected by various equilibrium processes, including the continuous exchange with blood, driven by osmotic and hydrostatic pressures. It is important to note that we are assessing baseline correlations across subjects, which does not imply that dynamic changes within individual subjects are uncorrelated between serum and dISF. Instead, this may indicate that partitioning between ISF and blood varies across individuals, so a high HSA value in serum does not necessarily correspond to a high HSA value in dISF.

### 3.4. Baseline Inflammatory Profile and NT-pBNP

Following a similar protocol, twenty volunteers were recruited to compare the baseline concentrations of inflammatory markers, as well as NT-pBNP, in serum and dISF. The primary aim was to validate the presence of valuable diagnostic markers in dISF, assess their levels, and examine the correlation between their distribution in dISF and serum.

The samples were analyzed for NT-pBNP, CRP, SAA, ICAM, and VCAM using Meso Scale Discovery (MSD) technology. Additionally, a panel of 45 well-established inflammation-related human protein biomarkers was screened using the Olink Target 48 Cytokine platform. The assays were conducted in compliance with scientific quality standards by qualified personnel at a GCLP and ISO 17025-certified facility at Firalis S.A. (Huningue, France) All calibration curves were fitted using Four Parameter Logistic (4PL) regression, and sample concentrations were back-calculated accordingly.

#### 3.4.1. V-Plex Injury Panel and NT-pBNP

**Figure 5:**
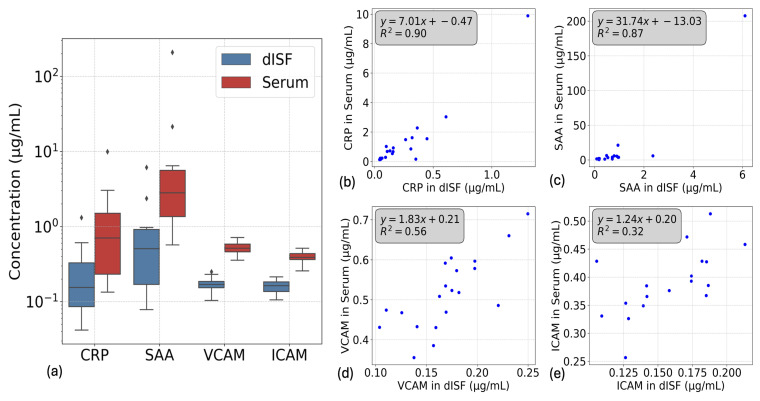
Comparison of inflammatory biomarker concentrations in serum and dermal Interstitial Fluid (dISF). (**a**) Boxplot representation of CRP, SAA, VCAM, and ICAM concentrations in serum (red) and dISF (blue), showing higher levels in serum across all biomarkers. (**b**) Correlation between CRP concentrations in dISF and serum, showing a strong linear relationship ($R^{2}=0.90$). (**c**) Correlation between SAA concentrations in dISF and serum, with a high correlation coefficient ($R^{2}=0.87$). (**d**) Correlation between VCAM concentrations in dISF and serum, with a modest correlation coefficient ($R^{2}=0.56$). (**e**) Correlation between ICAM concentrations in dISF and serum, with a low correlation coefficient ($R^{2}=0.32$).

The V-plex Injury Panel from MSD measured CRP, SAA, VCAM, and ICAM within the assay range for both dISF and serum. The concentration range of CRP in dISF was between 0.04 and 1.3 μg/mL, with a median concentration of 0.15 μg/mL (Figure 5a,b). In serum, CRP concentrations ranged from 0.15 to 10 μg/mL, with a median value of 0.7 μg/mL. The sample with 10 μg/mL of CRP in serum and 1.3 μg/mL in dISF was considered an outlier. The 10 μg/mL value is outside the physiological range and may indicate an infection [23]. With an R^2^ of 0.90, the correlation between CRP concentrations in dISF and serum was strong, but this high correlation was partially driven by the outlier. The slope of 7.01 indicates that CRP in dISF is approximately seven times more diluted than in serum, whereas the ratio of the medians suggests a dilution factor of 4.5.

The SAA distribution also demonstrated strong correlation (R^2^ = 0.87), but largely due to the presence of the same high-concentration outlier observed for CRP (Figure 5a,c). The slope of 31.74 suggests that SAA in dISF is roughly 31 times more diluted than in serum, while the ratio of the medians indicates a dilution factor of 5.5. The concentration range for SAA was 0.07 to 6 μg/mL in dISF and 0.56 to 207 μg/mL in serum, with respective median values of 0.5 μg/mL and 2.8 μg/mL (Figure 5a,c).

VCAM showed a moderate correlation between dISF and serum values, whereas ICAM showed little to no correlation (Figure 5d,e). Their concentration ranges were 0.1 to 0.25 μg/mL in dISF and 0.25 to 0.75 μg/mL in serum (Figure 5a), with respective median ratios of 2.4 (ICAM) and 3.0 (VCAM).

**Figure 6:**
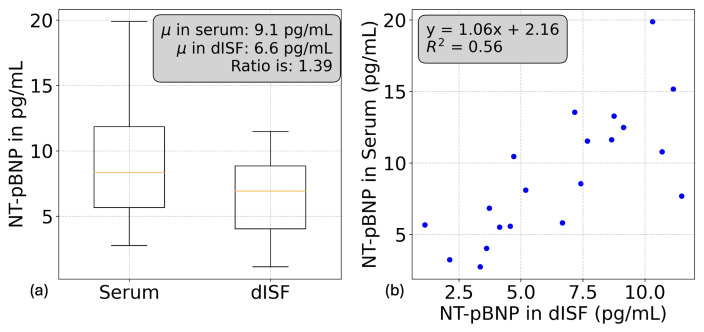
NT-pBNP dISF and serum comparison. (**a**) Boxplot and correlation analysis (**b**) of NT-pBNP concentrations, with a mean serum-to-dISF ratio of 1.39 and a moderate correlation ($R^{2}=0.56$) between NT-pBNP levels in dISF and serum.

Due to its low concentration range, NT-proBNP was measured separately using the R-plex MSD assay. The concentration range in dISF was 1.5 to 12 pg/mL and 2.5 to 20 pg/mL in serum (Figure 6a,b). The ratio between the medians was 1.39, and the R^2^ value was 0.56, indicating some correlation between dISF and serum.

Interestingly, the median ratios show a positive association with analyte size. Larger analytes exhibited higher median ratios and, consequently, greater dilution factors in dISF. Using approximate molecular weights of 8.5 kDa for NT-proBNP, 90 kDa for ICAM and VCAM, 115 kDa for CRP, and 250 kDa for SAA, we observed a relatively strong linear relationship between an analyte’s serum-to-dISF partitioning and its size ($R^{2}=0.87$). This observation aligns well with previous findings in the literature [24].

#### 3.4.2. OLink Target 48 Cytokine

**Figure 7:**
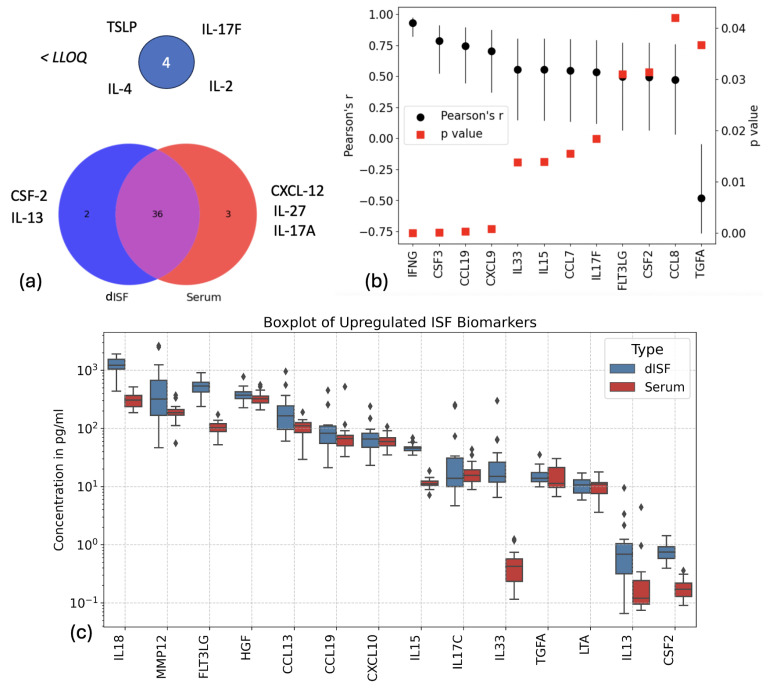
Comparison of Olink 48 Cytokines panel in serum and dermal interstitial fluid (dISF). (**a**) Venn diagram showing cytokines detected exclusively in dISF (blue), serum (red), and those common to both (purple). Cytokines below the lower limit of quantification (LLOQ) are indicated separately. (**b**) Pearson correlation coefficients (*r*, black dots) and corresponding *p*-values (red squares) for selected biomarkers in dISF and serum. (**c**) Boxplot representation of upregulated biomarkers in dISF compared to serum, highlighting differences in cytokine concentration levels.

Out of the 48 cytokines analyzed using the Olink Cytokine panel, 36 markers were detected above the limit of quantification (LOQ) in both dermal interstitial fluid (dISF) and serum (Figure 7a). Two cytokines, CSF-2 and IL-13, were exclusively detected in dISF, while three markers—CXCL12, IL-27, and IL-17A—were absent in dISF but measurable in serum. Additionally, four cytokines (IL-2, IL-4, TSLP, and IL-17F) were below the LOQ in both dISF and serum.

Among the 36 cytokines common to both matrices, 12 proteins demonstrated significant correlation between their concentrations in serum and dISF. Statistical analysis of these correlations was conducted using Pearson’s correlation coefficient, r, and associated *p*-values. The null hypothesis (H0) tested was: “the slope of the regression line is zero”. A biomarker was considered significantly correlated if the *p*-value was less than 0.05. Of these, four markers exhibited strong positive associations between dISF and serum concentrations, with r>0.7: IFN-γ, CSF-3, CCL19, and CXCL9 (Figure 7b). Interestingly, TGF-α showed a negative association, with r=−0.5, suggesting an inverse relationship between its levels in serum and dISF.

Five biomarkers were notably upregulated in dISF compared to serum: IL-18, MMP-12, FLT3LG, IL-15, and IL-33 (Figure 7c). These findings highlight the unique cytokine signature of dISF and suggest potential functional or regulatory roles of these biomarkers in the local tissue environment. Notably, all measured interleukins, with the exception of IL-27 and IL-1β, either displayed comparable levels in dISF and serum or were upregulated in dISF (Supplementary Materials). This analysis underscores the nuanced relationship between serum and dISF cytokine profiles, revealing both shared and matrix-specific markers that could serve as important indicators for local and systemic physiological or pathological states.

## 4. Discussion

This paper provides a comparison of serum and dermal interstitial fluid (dISF) across multiple analytes, revealing critical insights into their shared and distinct compositions. In the first study, based on 12 volunteers, we demonstrated the successful collection of dISF using a minimally invasive device, achieving an average yield of 3.4 μL per collection. Optical coherence tomography (OCT) imaging 48 h post-collection confirmed negligible risk of skin damage. Analysis of total protein content, human serum albumin (HSA), and immunoglobulin G (IgG) in serum revealed average concentrations of 70.89 g/L, 36.30 g/L, and 7.02 g/L, respectively. While these averages fall within physiological reference ranges, they are near the lower boundary of typical textbook values. Moreover, a significant number of individual serum samples showed HSA and IgG concentrations below the expected physiological limits, despite proper calibration and low variability in duplicate measurements. The origin of these lower values remains unclear but does not affect the correlation or the ratio between serum and dISF, as all samples were analyzed in the same assay run. The proportions of HSA and IgGs to total protein content align with the lower boundary of textbook reference values for HSA (50%) and IgG (10%) proportions. Interestingly, dISF showed similar protein proportions, with dISF/serum average ratios of 57% for total protein, 53% for HSA, and 47% for IgG. These values corroborate Reed’s hypothesis that ISF is an ultrafiltrate of plasma with protein concentrations between 50% and 60% of plasma levels.

Notably, total protein and HSA levels in dISF showed no correlation with their serum counterparts, whereas IgG displayed a strong correlation ($R^{2}=0.74$), primarily influenced by two high-value outliers. While chronic infections, autoimmune conditions, or recent vaccinations could potentially explain these elevated values, they remained within non-pathological ranges, and no definitive explanation could be identified.

It is crucial to emphasize that our analysis focuses on baseline correlations across individuals, which does not necessarily reflect the dynamic relationships between serum and dISF within the same individual. The lack of strong correlations between serum and dISF analyte levels may indicate that the partitioning of proteins between these compartments is influenced by inter-individual variability. For instance, a high HSA concentration in serum does not always correspond to a proportionally high HSA level in dISF. This observation challenges the notion of a straightforward or uniform partitioning process and suggests a more complex interplay of factors regulating the exchange between blood and ISF.

In the second study, involving 20 volunteers, we analyzed CRP, SAA, VCAM, ICAM, and NT-proBNP concentrations in both dISF and serum. CRP and SAA were significantly diluted in dISF, with median dilution factors of 4.5 and 5.5, respectively. While both markers showed strong correlations with their serum counterparts ($R^{2}=0.90$ for CRP and $R^{2}=0.87$ for SAA), the correlation for CRP was only partially influenced by a high-value outlier, whereas the correlation for SAA was largely driven by the same outlier. VCAM showed a modest correlation with serum levels, while ICAM exhibited little to no correlation. NT-proBNP, in contrast, demonstrated a moderate correlation ($R^{2}=0.56$) and a median dilution factor of 1.39. A key observation was the relationship between molecular size and analyte dilution in dISF, with larger proteins exhibiting higher dilution factors. This trend is consistent with the hypothesis that molecular size governs passive transport and partitioning between blood and the interstitial space [24].

Interestingly, the “pathological outlier” with elevated CRP and SAA levels was detectable in dISF, highlighting its potential as a proxy medium for diagnostic applications. This finding underscores the capacity of dISF to reflect pathological states, offering a minimally invasive alternative for monitoring biomarkers associated with systemic conditions.

Furthermore, the detection of NT-proBNP at comparable levels in both dISF and serum suggests that dISF NT-proBNP concentration may serve as a reliable proxy for blood NT-proBNP levels. This opens promising avenues for the use of dISF in the non-invasive diagnosis and monitoring of cardiac failure.

Using the Olink 48 Cytokine panel, we quantified 36 out of 45 cytokines above the LOQ in both dISF and serum. Notably, CSF-2 and IL-13 were uniquely detected in dISF, while CXCL12, IL-27, and IL-17A were only measurable in serum. Among shared cytokines, 12 exhibited significant serum-dISF correlations, with strong positive associations (r > 0.7) for IFN-γ, CSF-3, CCL19, and CXCL9. Conversely, TGF-α showed a negative correlation (r = −0.5), suggesting an inverse relationship between serum and dISF levels. Five cytokines (IL-18, MMP-12, FLT3LG, IL-15, and IL-33) were upregulated in dISF, highlighting their potential tissue-specific roles. Most interleukins showed comparable or elevated levels in dISF, except for IL-27 and IL-1β. Our findings align partially with previous studies. For example, similarities with the work of Sjöbom et al. [13] in synovial blister fluid (SBF) include the absence of IL-17A in dISF and the strong positive association of IFN-γ and CXCL9. However, the negative association of TGF-α in dISF contrasts with its strong positive correlation in SBF, warranting further investigation to determine whether these differences stem from matrix-specific effects. Similarly, comparisons with Li et al. [14] revealed consistent FLT3L upregulation between fracture blister fluid (FBF) and dISF, but other cytokine trends varied.

Beyond physiological characterization, this study provides practical insights for the development of wearable biosensors targeting dermal interstitial fluid. The detection of CRP, SAA, VCAM, ICAM, NT-proBNP, and more than 35 other cytokines in dISF at measurable concentrations confirms their relevance as potential targets for non-invasive monitoring. Establishing their correlation profiles with serum further informs biomarker selection, helping to prioritize candidates with meaningful clinical translatability. In addition, the quantification of total protein content and the confirmed presence of key analytes such as human serum albumin (HSA) in dISF offer valuable guidance for designing calibration and testing solutions. This knowledge supports the creation of physiologically relevant test matrices that reflect the biochemical complexity of dISF, enabling more accurate assessments of biosensor specificity and selectivity during development.

## 5. Conclusions

The reported experimental findings shed light on the intricate relationships between serum and dISF, in terms of key biomarkers and their dilution, revealing similarities in the composition of their main constituents, as well as shared analytes and unique profiles. Our results underscore the significant role that molecular size plays in influencing the partitioning between these two matrices. They also reveal a more complex interplay of factors regulating the exchange between blood and ISF beyond concentration alone. To the best of our knowledge, this work is the first to report quantitative measurements of protein inflammatory biomarkers in dISF and to investigate their relationship with serum levels. The detection of cytokines, NT-proBNP, and other clinically relevant biomarkers in dISF—including the identification of a “pathological outlier” with elevated CRP and SAA levels—highlights its potential as a minimally invasive proxy for diagnostics based on wearables and point of care devices exploiting dISF. Interestingly, NT-proBNP originally exhibited comparable concentrations in both compartments, suggesting that dISF could serve as a reliable surrogate for blood-based NT-proBNP measurements. Altogether, these findings establish dISF as a promising matrix for clinical applications. Future research should focus on characterizing the dynamic interplay of analyte levels between dISF and serum in both healthy and pathological conditions, paving the way for the development of minimally invasive biosensors.

## Data Availability

Data are contained within the article.

## Supplementary Materials

The following supporting information can be downloaded at: https://www.mdpi.com/article/10.3390/bios15050301/s1, Figure S1: Comparison of interleukin concentrations in dermal interstitial fluid (ISF) and serum, measured using the Olink^®^ Target 48 Cytokine panel. Each box represents the distribution of interleukin levels (in pg/mL) on a logarithmic scale. ISF (blue) and serum (orange) values are shown for each biomarker.

## Abbreviations

The following abbreviations are used in this manuscript:

dISF	Dermal Interstitial Fluid
SBF	Suction Blister Fluid
HSA	Human Serum Albumin
IgG	Immunoglobulin G
CRP	C-Reactive Protein
SAA	Serum Amyloid A
VCAM	Vascular Cell Adhesion Molecule 1
ICAM	Intercellular Adhesion Molecule 1
NT-pBNP	N-terminal pro b-type natriuretic peptide
CGMs	Continuous Glucose Monitors
PEA	Proximity Extension Assay
MSD	Meso Scale Discovery
OCT	Optical Coherence Tomography
BCA	BiCinchoninic acid Assay
