# Supplementary material for: Bridging Blood and Skin: Biomarker Profiling in Dermal Interstitial Fluid (dISF) for Minimally Invasive Diagnostics
Source: Biosensors (Basel). 2025 May 9;15(5):301. doi: 10.3390/bios15050301 (PMC12110442; doi:10.3390/bios15050301)
Supplement: Supplementary file 1 [file biosensors-15-00301-s001.zip › biosensors-3598259-supplementary.pdf]

Supplementary material:

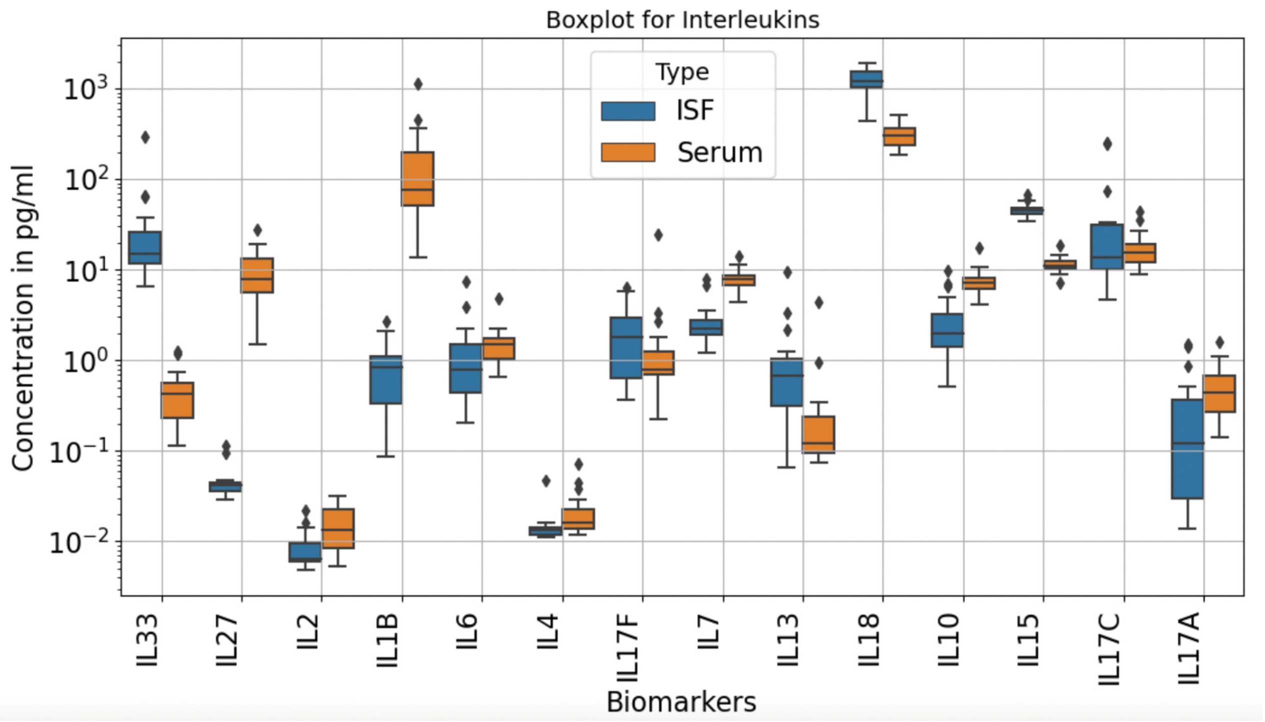

Figure S1: Comparison of interleukin concentrations in dermal interstitial fluid (ISF) and serum, measured using the Olink® Target 48 Cytokine panel. Each box represents the distribution of interleukin levels (in pg/mL) on a logarithmic scale. ISF (blue) and serum (orange) values are shown for each biomarker.
